# Understanding yeast shells: structure, properties and applications

**DOI:** 10.5599/admet.2118

**Published:** 2024-02-15

**Authors:** Larissa Silva de Macêdo, Samara Sousa de Pinho, Anna Jéssica Duarte Silva, Ingrid Andrêssa de Moura, Benigno Cristofer Flores Espinoza, Maria da Conceição Viana Invenção, Pedro Vinícius Silva Novis, Marco Antonio Turiah Machado da Gama, Matheus do Nascimento Carvalho, Lígia Rosa Sales Leal, Bruna Isabel Santos Cruz, Beatriz Mendonça Alves Bandeira, Vanessa Emanuelle Pereira Santos, Antonio Carlos de Freitas

**Affiliations:** Laboratory of Molecular Studies and Experimental Therapy - LEMTE; Department of Genetics, Biosciences Center, Federal University of Pernambuco; Pernambuco - Recife 50670-901, Brazil

**Keywords:** Nanocapsules, delivery vehicle, β-glucans, encapsulation, immunostimulator

## Abstract

**Background and purpose:**

The employment of yeasts for biomedical purposes has become increasingly frequent for the delivery of prophylactic and therapeutic products. Its structural components, such as β-glucans, mannan, and chitin, can be explored as immunostimulators that show safety and low toxicity. Besides, this system minimizes antigen degradation after administration, facilitating the delivery to the target cells.

**Review approach:**

This review sought to present molecules derived from yeast, called yeast shells (YS), and their applications as carrier vehicles for drugs, proteins, and nucleic acids for immunotherapy purposes. Furthermore, due to the diversity of information regarding the production and immunostimulation of these compounds, a survey of the protocols and immune response profiles generated was presented.

**Key results:**

The use of YS has allowed the development of strategies that combine efficiency and effectiveness in antigen delivery. The capsular structure can be recognized and phagocytized by dendritic cells and macrophages. In addition, the combination with different molecules, such as nanoparticles or even additional adjuvants, improves the cargo loading, enhancing the system. Activation by specific immune pathways can also be achieved by different administration routes.

**Conclusion:**

Yeast derivatives combined in different ways can increase immunostimulation, enhancing the delivery of medicines and vaccine antigens. These aspects, combined with the simplicity of the production steps, make these strategies more accessible to be applied in the prevention and treatment of various diseases.

## Introduction

Initially described for their use in the food industry, yeasts such as *Saccharomyces cerevisiae* have characteristics, such as fermentation capacity and adaptability, that make them biotechnological platforms with a broad range of applications [1-3]. Thus, it did not take long for its functionality to be explored by the pharmaceutical industry, ranging from the production of vaccines and therapeutic proteins to the delivery of drugs and vaccine antigens [1,4,5]. Its use as an antigen carrier has received special attention due to its ability to protect against physiological degradation, favor antigenic presentation, and present adjuvant properties [6,7].

In addition to the whole yeast cell as a carrier and immunomodulator, some derivatives of its structure have also been extensively studied [8-10]. The yeast shells (YS) are structures obtained from intact cells, taking advantage of the natural predisposition to assimilate different molecules. These YS are generated through treatments with physicochemical agents that can favor their permeability to molecules and the ability to interact with target cells. The yeast nuclear, cytoplasmic, and membrane structures are removed, resulting in a scaffold composed of β-glucans, mannan, and chitin [1]. The resulting structure can incorporate the material of interest, like performed by the liposomal shells also adopted as a delivery system, and further be administered via parenteral or oral [11].

The surface carbohydrates of the particles are recognized by receptors expressed in macrophages and dendritic cells, leading to internalization by phagocytosis [12]. This specific antigen delivery, even to remote sites, is interesting in treating diseases with inflammatory characteristics [13,14]. The influence of inflammatory processes on the progression of chronic diseases is already well established, and the use of yeasts and their derivatives has been studied to propose more effective and specific treatments targeting diseases such as diabetes, obesity, osteoarthritis, and cancer [14-17].

Despite the advantages presented, some parameters need improvements, such as the lack of standardization in the incorporation process, which influences the reproducibility of the protocols, as well as limited information about adequate quantification of the cargo. Even though the proof of concept about this delivery system has already been demonstrated, greater technical detail is necessary for clinical translation. In this way, understanding targeted studies can be the key to better use of YS.

Throughout this review, we intend to give a general overview of yeast capsules, from their structural characterization and adjuvant properties to their application as a delivery system for drugs and vaccine antigens that occur through various routes of administration. Here, we will also address some of the main protocols adopted to obtain capsules to point out possible ways to work with this delivery system. Part of the current challenge in establishing this platform is precisely the standardization and knowledge about the concentration and quantity of cargo that can be carried, combining it with the best method for each type of molecule to be delivered. The present review, besides compliance protocols and the main results of yeast shell studies, can provide insights to overcome the bottlenecks that still limit the use of this delivery system.

## Characterization and properties of yeast shells

**Figure 1.**
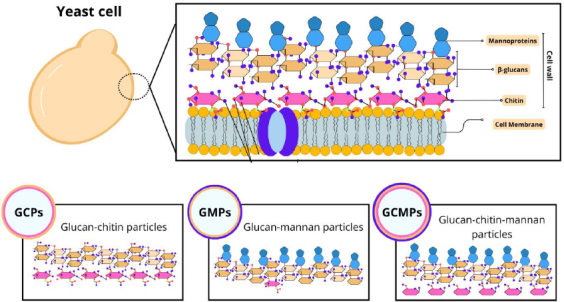
Representation of different yeast shells based on composition: glucan-chitin particles (GCPs), glucan-mannan particles (GMPs), and glucan-chitin-mannan particles (GCMPs).

Among the different studies involving particles derived from yeast, there is a diversity in the nomenclatures adopted. For example, capsules composed of β-glucan can be referred to as β-glucan particles (GPs), whole glucan particles (WGP), or even yeast cell wall particles (YCWP). Furthermore, some variations in composition can be found, such as glucan-chitin particles (GCPs), glucan-mannan particles (GMPs), and glucan-chitin-mannan particles (GCMPs), as shown in Figure 1. The protocol of preparing these particles influences their composition, size, and, consequently, the most appropriate terminology [18].

In this study, we will adopt the term YS to refer to capsules derived from yeast to address general aspects. Furthermore, as it is a common component of all particles, most studies refer to β-glucan structures and the influence of this component on transport and interaction with target cells. Therefore, we will focus on their characteristics, preparation, and applications.

### Cell wall components

The yeast cell wall is composed of polysaccharides such as β-glucans, chitin, and mannoproteins that favor its rigidity and thickness [19]. It also acts on physical protection and cellular osmotic maintenance [20]. The composition and distribution of cell wall components can vary between yeast species. In *S. cerevisiae*, for example, the cell wall comprises about 30% of the dry-weight cell and is structurally defined by internal and external layers [21].

β-glucans present different structural and physicochemical variations depending on their origin (yeast, bacteria, cereals), influencing their physiological functions [22]. In yeast, they represent the main component of the internal layer, with high molecular weight and degree of branching. It consists of a β-1,3-glucan backbone polymeric structure with an amorphous β-1,6-glucan side chain. Functionally, β-Glucan is an immune activator of the innate immune response that can act nonspecifically and specifically on the immune system with PAMPs (pathogen-associated molecular patterns) [23,24]. Therefore, GPs can be used for enteral transport of probiotics [25] and delivery of medicines and vaccines [26].

Chitin is a minor constituent in the internal layer, and its crystalline structure provides resistance to cell wall stretching and fiber insolubility [27,28]. Its arrangement can be seen in free form or linked to the β-1-3-glucan or β-1-6-glucan portions, functioning as an essential component for normal cell division [29,30].

In the outermost layer, mannoproteins are present, which consist of mannose residues linked by glycosidic bonds [31]. This fibrillar layer is characterized by the presence of electrons that confer the yeast's surface anionic charge through the phosphorylation of the mannosyl side chains [27,28]. Furthermore, mannoproteins are associated with cell-cell recognition events [20]. Some studies have revealed that β-glucan and mannan of the yeast cell wall can activate macrophages, thus promoting the organism's non-specific immune response [24,32,33]. However, studies with Candida albicans have demonstrated that mannans mask β-1-3-glucan molecules from dectin-1 receptors, contributing to yeast evasion of innate immunity [34-36]. On the other hand, for yeasts such as *S. cerevisae*, *K. lactis* and *P. pastoris*, which have GRAS status, their viability does not have the same characteristics and, in addition, inactivation by heating increases the exposure of β-glucans in these species [6]. Figure 1 demonstrates the structure of the yeast cell wall.

### YS general features

Yeast shells are biodegradable, porous, and hollow, allowing the carrying of nucleic acids, antigens, adjuvants, drugs and other molecular structures with targeted delivery and protection against degradation [18,37-42]. The size can vary from 2-4 μm, which enables specific phagocytosis by dendritic cells and macrophages [43,44]. Due to the porous characteristic of the capsule, there is the possibility of the product leaking, which can be overcome by coating with cationic chitosan and anionic alginate, forming a matrix that protects the pores [18,45]. Other strategies that can be used to solve this problem are preloading the capsules with anthocyanins and sequential deposition of polysaccharides with opposite charges, such as chitosan and chondroitin sulfate [46], another study used colloids of aluminum hydroxide to form aggregates with the transported molecule of interest within the capsule [38]. Furthermore, stearin blocking can be done and chitosan, tripolyphosphate and alginate can be used to form colloidal particles capable of keeping the molecules transported inside the particle [42,47].

Structures derived from yeast have gained prominence in the vaccine development and delivery of nanomedicines administered orally, maintaining the integrity of the carried molecule. The composition of the capsules makes them resistant to pH and gastric enzymes, allowing them to pass through the gastrointestinal tract where they interact with M cells that will mediate biodistribution to components of mucosal immunity [13,48].

Furthermore, YS can be constructed by conjugating β-glucans with other molecules to improve delivery, as performed by Chowdhury *et al.* [49]. They synthesized a vehicle composed of taurocholic acid and β-glucan, capable of protecting molecules from the harmful environment of the stomach and accelerating the absorption of particles by the circulatory system, in addition to having target specificity for the liver, the organ of interest in the study. An important aspect of the technology developed from YS is the ability to form encapsulated cores in situ within the hollow cavity. Thus, antigens can be incorporated and combined by the layer-by-layer (LbL) self-assembly methodology of nanomaterials, which are held together by electrostatic interactions [8]. The formation of stable complexes between negatively charged genetic material and cationic polymers is the most commonly studied approach to developing nonviral delivery agents. Soto and Ostroff [8] developed an approach to create nanoparticle materials in order to protect and deliver DNA to phagocytic cells efficiently. In this way, the main barriers found in the transport of nucleic acids, such as low protection of the material with a consequent reduction in payload, inefficiency in delivery, release and uptake by cells, can be overcome using this system. The delivery system demonstrated efficacy in DNA delivery and transfection of both macrophages and dendritic cells through in vitro and in vivo evaluations [8].

In this sense, new strategies for drug and vaccine delivery vehicles have advanced, and it is worth investigating the use of fungal β-glucans as materials for capsule synthesis, whether or not these are conjugated with other materials [50,51].

### Preparation of yeast shells

The methodologies used in studies to obtain the different YS have some similarities [10,52-54]. The extraction and purification process of these yeasts involve some steps to guarantee the purity and effectiveness of the YS without altering their morphology [3,6,54]. YS is obtained through the elimination of nucleic acids and some intracellular proteins present in the yeast cells, resulting in hollow particles capable of carrying drugs, antigens, and proteins. This procedure employs a simple treatment with alkaline and acid pH, associated with a heat treatment, using NaOH (80 °C) and HCl (60 °C) [3,10,52-54]. After carrying out this approach, the only remains of the yeast cells are β-glucans, the predominant component of the cell walls of these yeasts, and chitin, lipids and proteins in smaller proportions [18,43]. Young *et al.* [55] verified the structural composition of purified YS from *S. cerevisiae* that contained ~80 % branched 1→6-β, 1→3-β-glucan, 2 to 4 % chitin and <1 % mannan. However, the composition of each component can be changed after extraction of these particles, depending on the strain or growth stage. For example, the composition of chitin in the cell wall can vary from 0 to 36 % depending on the growth stage [55]. Other aspects, such as the number of cells, temperature, concentration and volume of solutions, vary between different studies [52,56].

**Figure 2.**
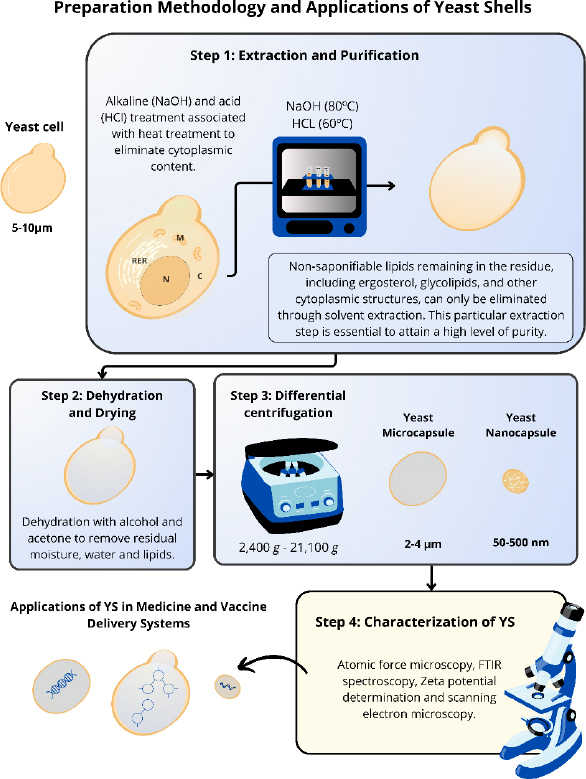
Purification and characterization of YS from yeast. Schematic representation of the methodology used in the studies to obtain the YS.

Subsequently, these yeast shells are dehydrated with alcohol and acetone to remove residual moisture and improve the YS porosity. Then, the particles are dried to obtain a solid and thermostable form of the YS [10,53,54,57] (Figure 2).

Some aspects, such as temperature, concentration, and volume of solutions, vary among the different studies. The induction of variations in the final size of the YS was proposed by Xu *et al.* [58]. Through differential centrifugation, with a range of rotations from 2,400*g* to 21,100*g*, different sizes of nanoparticles derived from YS (NPY) can be obtained, ranging from 50 to 500 nm (Figure 2). For YS nanocapsules obtainment, it is necessary to break these structures through high-speed centrifugation to reduce the size of the original particle. The reduction of these particles demonstrated an improved anticancer effect compared to larger particles.

The standardization of this process enables its reproducibility with greater accuracy. Furthermore, this methodology offers a cost-effective approach to large-scale production of YS, making them more accessible than other delivery systems. After the preparation, it is necessary to check the particle morphology, size, uniformity, and stability. Most studies employ atomic force microscopy, FTIR spectroscopy, Zeta potential determination, and scanning electron microscopy to characterize these YS [38,58,59].

The subsequent steps to allow the entry of these materials into these YS vary according to the objectives proposed in these studies. The drug or antigen to be carried may be contained, uniformly dispersed or chemically linked to the GPs [3]. Pan *et al.* [40] used ovalbumin (OVA) as a model antigen conjugated to the YS surface. By incubation with YS oxidized with NaIO_4_. Through chemical treatment, OVA was attached to the surface of YS, forming YS-OVA particles with a drug loading of 20 %. The results showed that YS facilitated antigen absorption by dendritic cells, in addition to stimulating maturation and inducing the release of immune co-stimulatory molecules by DCs. Thus revealing the potential use of YS in the administration of antigens carried on its external surface [40]. Sabu *et al.* [60] demonstrated the efficiency of an oral delivery system for YS-carrying insulin, using 100 mg of YS added to a volume of 10 mL of insulin with a concentration of 100 μg/mL in HCl 0.01 N. This material was agitated at 400 rpm for 3 hours and then centrifuged at 2,000 rpm for 10 minutes. This approach demonstrated encapsulation efficiency (EE) of 89.84±1.04% [60].

Another study using these particles as carriers addressed YS encapsulating curcumin, employing a stock solution of curcumin (0.2 mg/mL) in ethanol. This preparation was added to the dried YS and mixed with UltraTurrax T10 (IKA) to create a uniform distribution of curcumin within the YS. The EE of the composites prepared with three measures of YS compound with 0.55, 0.11 and 0.055 wt.% curcumin showed that the real value of the mass fraction of curcumin with 0.621±0.064, 0.139±0.007 and 0.071±0.004 % respectively, was slightly higher than planned. This approach allowed the encapsulation of curcumin, potentially increasing its stability and bioavailability in pharmaceutical applications [52]. Both studies demonstrated potential applications of YS in drug delivery systems [52].

Although numerous studies demonstrate efficient transport by YS, when using a porous particle as a delivery systems, transported substances may be lost during their delivery to antigen-presenting cells (APCs) due to external environmental conditions [45]. Given this, recent methodologies propose the application of YS associated with adjuvants or stabilizing agents. Zhu *et al.* [38] used YS associated with aluminum chloride (AlCl_3_) to carry a vaccine against human toxoplasmosis. The particles were incubated in a solution containing 0.125 M AlCl_3_ for 2 hours. Subsequently, the excess aluminum salt outside the YS was removed by centrifugation. To further optimize the formation of aluminum hydroxide colloids in the YS, the solution was resuspended using 2.0 mL of ammonium hydroxide (0.05 M), followed by 10 minutes of stirring to facilitate precipitation of the aluminum salt. Finally, ultrasonography was performed for 30 minutes at a frequency of 25 kHz and power of 100 W to ensure the formation of stable aluminum hydroxide colloids within the YS [38].

A similar methodology was reproduced by Liu *et al.* [53] to obtain NPY with aluminum for use as a vaccine delivery system against hepatitis B. The study used GP-Al (5.0 mg/mL, 1 mL) mixed with HBsAg proteins (10 mg/mL, 25 μL) stirred at room temperature for 30 minutes. After that, the residue was resuspended in PBS and subjected to three centrifugations to form GP-Al-HBsAg particles. The vaccine showed promising results in improving humoral and cellular immune responses, representing a safe and promising system for antigen delivery [53]. More information on payload strategies and encapsulation efficiency through YS is summarized in Table 1.

**Table 1 -:** Payload trapping strategies.

Payload class	Payload trapping molecules	EE, %	Load levels and YS	Payload location	Ref.
Peptide - protein	Polyethyleneimine (PEI) / cationic polymer	68	45 ng/μl fetal calf serum (FCS); 0.2 % PEI; 1 mg of YS	Within the hollow shell	[18]
DNA- plasmid	Agarose / anionic polymers	>80	500 mg aliquot of YS; 500 ul of DNA in agarose at 50	Within the hollow shell	[18]
Peptide - protein	Sodium periodate (NaIO_4_) / anionic polymers	-	50 mg of YS; 1,5 mL of NaIO_4_ solution_;_ OVA protein solution and aldehyde YS were mixed at the mass ratio of 2:1	Linked to the surface YS	[40]
Small molecule - water insoluble- doxorrubicina (DOX)	Chitosan / cationic polymer and alginate / anionic polymers	52.4	5 mg of YS; 100 μL of DOX solution (1 mg/mL); chitosan (0.4 % w/v); TPP/alginate solution (1.0 mg/mL of TPP, 0.4 mg/mL of alginate sodium)	Within the hollow shell	[45]
RNA- tRNA and DNA- sodium salt from Salmon testes (DNA)	PEI / cationic polymer	>85	YS (10^8^ particles/mL); DNA or tRNA (10 mg); PEI (3 mg/mL)	Within the hollow shell	[8]

## Yeast shells as adjuvants and carriers in biomedical therapies: studies and perspectives

Microcapsules and nanocapsules derived from glycoproteins or cell walls are formulated for biomedical applications, taking advantage of the immunostimulatory properties of yeast. Therefore, they play fundamental roles as adjuvants, immunomodulators, and carriers for the delivery of therapeutic agents [4,11,61]. Yeast cell wall components play a crucial role in mechanical resistance, cell adhesion, and modulation of the host immune response [11,62].

β-glucans (β-1,3 and β-1,6) are readily recognized by receptors in the host body, such as dectin-1 and complement receptor 3 (CR3), leading to opsonization, inflammatory cell influx, and immune responses T cells (Th1, Th17, and cytotoxic) [2,63-65]. Mannan and chitin of yeast shells also bind to pattern recognition receptors, inducing characteristic T cell responses, depending on the proportion between these components [66,67].

The ability of yeast shells to activate several T cell response pathways, including those induced by antibodies (Th2 type), makes them promising adjuvants and carriers in disease therapies where treatment efficacy and immunological stimulation act synergistically [6,11,68]. For example, in research into vaccines against viral, bacterial, fungal, and protozoal diseases, yeast shells have been employed as adjuvants and carriers to amplify T-cell and antibody-mediated immune responses. Soares *et al.* [69] found that the use of YS as an adjuvant in a hepatitis B vaccine triggered the release of antigen-specific Th1, Th2, Th9, Th17, Th22, and Treg-related cytokines, promoting antiviral immunity [69]. Otherwise, Liu *et al.* [53] created hybrid aluminum and β-glucan microspheres to deliver HBV antigens to enhance humoral and cellular immune responses [53]. For fungal infections, glucan-chitin particles were used as adjuvant carriers against lung infection caused by *Coccidioides posadasii*, being able to stimulate a mixed Th1 and Th17 response and improve the protective response against infection [70]. Furthermore, as new strategies to enhance the activation of protective CD8+ T cells in a coccidioidomycosis vaccine, Cole *et al.* [71] added Endo-Porter peptides to increase the release of proteins into the cytoplasm of APCs after phagocytosis of GPs [71]. As a hybrid carrier of GP-AS04 in a vaccine against *Toxoplasma gondii*, it mediated the delivery and facilitated the uptake of antigens by increasing the secretion of pro-inflammatory cytokines [38].

Regarding cancer therapies, studies have investigated YS to stimulate immune responses targeting tumor cells. The potential of YS to activate cytotoxic T lymphocytes and their ability to transport cancer-specific antigens can lead the immune system to identify and destroy cancer cells [72,73]. To prevent the development of tumors, NPY modifies the immunosuppressive microenvironment in tumors and tumor-draining lymph nodes [58]. Jing *et al.* [54], for example, developed a broad-spectrum therapeutic vaccine with neoantigens coupled to YS capable of inhibiting tumor growth in cancer cell models (EG7·OVA, B16F10, 4T1, and CT26) [54]. In addition, studies have also investigated the impact of yeast shells on the modulation of macrophage phenotypes, particularly the transition from M2 to M1 macrophages, as this transition is known to play a key role in influencing cancer progression [74-76]. Yeast-derived whole β-glucan particles (WGPs) have been identified as potent inducers of macrophage polarization. They can drive M0 macrophages towards the M1 phenotype, characterized by pro-inflammatory and antitumor properties [74]. Moreover, WGPs have demonstrated the ability to convert existing M2 macrophages and tumor-associated macrophages (TAMs) into the M1 phenotype [74,75]. This unique capability positions yeast-derived WGPs as promising agents for cancer immunotherapy, not only by directly affecting tumor cells but also by reshaping the tumor microenvironment through the conversion of immunosuppressive macrophages into pro-inflammatory M1 macrophages [76]. In autoimmune diseases, where immune regulation is compromised, the use of yeast shells as adjuvants has been studied as a way to rebalance immune responses. Furthermore, they can be used as delivery systems for genetic material in oral vaccines, allowing the introduction of therapeutic genes into specific cells, increasing efficacy, and improving systemic mucosal immunity [6,11,77]. Hu *et al.* [12] used TNF-α RNAi mediated by oral yeast microcapsules in the therapy of rheumatoid arthritis, the results of which indicated a reduction in pro-inflammatory factors and a greater systematic regulation of the inflammatory response [12].

Another recently developed application using capsules demonstrates the combination with micro/nanorobot technology, administered as therapeutic molecules or medications in a targeted manner. Furthermore, they have considerable promising *in vivo* applications related to inflammation of the gastrointestinal tract due to the adoption of biocompatible endogenous fuels [78]. In the study by Zhang *et al.* [78] capsules derived from the yeast *S. cerevisiae* were used as a biocompatible and intelligent material to design self-adaptive micro/nanorobots with dual biomotor (TBY robot) to reach multiple biological barriers, allowing active administration of drugs for therapy of the TGI. In a murine animal model, TBY robots attenuated inflamemation and improved disease pathology. TBY robots exhibited enzymatic actuation and the capacity to reprogram macrophages, enabling autonomous adaptation to changes in the surrounding environment to penetrate multiple biological barriers to reach inflamed sites. These effects ensure a precise therapy for gastrointestinal inflammation [78].

Therefore, as new research and advancements occur in the area of yeast derivatives, the potential of these capsules as facilitators of innovative therapies continues to grow, paving the way for promising future developments and applications. However, different types of yeast-derived particles offer varied approaches to meet the specific needs of each application, and distinct delivery routes can influence their action as a carrier and enhancer of the immune response [14,79].

## Possibility of molecules carried by YS

Both yeast and yeast shells can transport a broad spectrum of molecules such as DNA, mRNA, iRNA, and proteins (Table 2). On the other hand, the structure and composition of YS allow for the additional advantage of incorporating and carrying nanoprobes (performing contrast-enhanced imaging exams) and nanoparticles loaded with vaccines or medicines [13].

**Table 2:** Diversity of yeast-derived molecules and structure types that can be used for carriage.

Target-disease	Molecule	Capsule type	Ref.
HFD-obesity	shRNA	Microcapsule	[3,17,18]
Cancer	Protein	β-Glucan whole particle	[3,16,18]
-	DNA associated to tRNA and PEI polymers	Yeast cell wall particle (YCWP)	[8]
*Cryptococcus neoformans*	Protein extract	β-glucan particles	[61]
*Coccidioides posadasii*	Protein	Capsules formed with different glucan-chitin concentrations	[70]
*Cryptococcus neoformans*	Protein	β-glucan particles with a silica structure using tetraethylorthosilicate	[80]
Cepa KN99 do *C. neoformans*	Aggregated nickel particles	β-glucan particles	[81]
-	Pseudovirus	Yeast cell wall particle modified with positively charged polyethyleneimine	[82]
-	Quantum dots	β-glucan particles	[3,13,18]
-	Iron oxide nanoparticles	β-glucan particles	[13]
-	Fluorescent organic nanoparticles	β-glucan particles	[13]
Inflammatory diseases	Nonsteroidal anti-inflammatory medication	β-glucan particles	[13]
Cancer	Chemotherapy agent	β-glucan particles	[13]

YS has been used to deliver nucleic acids or protein subunits as an oral vaccine strategy [11]. Aouadi *et al.* [83] employed this system to silence the expression of Map4k4 and inhibit the production of TNF-α using 40 pmol of siRNA in 10^8^ GeRP40 (β-1,3-D-Glucans). They proposed an oral vaccine model against inflammatory diseases, such as rheumatoid arthritis, ankylosing spondylitis, Crohn's disease, and psoriasis [83]. An *in vitro* study carried out by Plavcová *et al.* [52] observed that the treatment of THP-1 cells (555,555 cells/mL) with 10 μL of YS/curcumin promoted a decrease in NF-κB/AP-1 activity. This model acts as an antioxidant, which could eventually be used for inflammatory diseases [52].

Regarding the concentration of the target compound to be carried by YS, the specific amounts are not well described for each application. However, some studies have demonstrated different quantities that enable distinct applications of these structures, as reviewed in Table 3.

**Table 3:** Antigens loaded in different models of yeast capsules, concentrations, and their applications.

Model	Content of GP particles	Loaded antigen	Administration	Ref.
C57BL/6 mice	500 μg	Extract of *Toxoplasma gondii*	Oral	[38]
C57BL/6 and BALB/c mice	(0,375 mg/kg) of NP YCW in 50 μl	-	Intratumoral	[58]
C57BL/6 mice	30 mg of microcapsule per kg	IL-1β shRNA	Oral	[14]
THP1 XBlue™ MD2 CD14 cell line	670 μg/ml	Curcumin	-	[52]
C57BL/6 mice	1 mg	OVA	Subcutaneous	[84]
ApoE -/- mice	100 mg of YC in 10 ml	Rapamycin (RAP)	Oral	[85]
BALB/c mice	2×10^8^ YC	Indomethacin (IND) and paclitaxel (PTX)	Oral	[13]
B6.V -Lep ob /J mice	1 mg/ml	ARNip	Intraperitoneal	[86]
C57BL/6 mice	50 mg of YS	OVA	Subcutaneous	[40]
C57BL6/J mice	10^8^ YC	iRNA	Oral	[83]
NIH3T3-D1 cell line	8 μg of DNA/ 10^6^ YC	gWizGFP plasmid	Transfection	[8]

## Administration routes for yeast shells

An important point to consider for the efficiency of using capsules for the purposes presented above is the route of administration. Different approaches employ oral, intranasal, intradermal, subcutaneous, and intraperitoneal routes, with advantages and disadvantages further discussed in this section.

The size of the carrier structure can influence the choice of the most appropriate route of administration. Some studies suggest that the diameter of yeast cells, ranging from 1 to 10 μm, can lead to local adverse events when administered intramuscularly or intraperitoneally [59]. Adverse reactions would be related to the local inflammatory response due to the accumulation of yeast due to low absorption. This effect could be minimized or eliminated by employing yeast-derived capsules (diameter of 2 to 4 μm) not associated with exacerbated local inflammatory processes. Furthermore, microcapsules stand out for their low toxicity to the body, enabling the administration for long periods without causing damage to the biological system [87].

Subcutaneous injection was adopted in a murine model to evaluate antitumor efficacy and induced Th1/Th17 immune responses, characterized by the production of IL-17, and inhibited tumor growth by up to 91.8 % [88]. The intraperitoneal route was used in a β-glucan-based delivery system for vaccination against SARS-CoV-2, showing a robust cellular and humoral immune response [89]. In a randomized clinical trial using a dendritic cell vaccine with YS for stage III/IV melanoma with intradermal application, there was an improvement in overall survival with no difference in progression-free survival [90].

For oral administration, it is necessary to consider the challenges related to low pH, bile salts, and digestive enzymes [91], besides ensuring adequate tissue absorption and targeting [11]. The use of YS overcomes most of these adversities. When used as a shRNA delivery vehicle in post-traumatic osteoarthritis gene therapy, YS passed through the gastrointestinal tract without degradation after oral administration [92]. Similar findings were reported to reduce parasite load in the murine model of visceral leishmaniasis [93].

Concurrently, using orally administered WGP β-glucans in conjunction with monoclonal antibody therapy against tumors, a significant tumor regression of ≥80 % was achieved compared to treatment with monoclonal antibodies alone. In this case, WGP is processed by macrophages that prime the leukocyte complement receptor 3 (CR3), allowing these cells to eliminate opsonized tumors with the complement fragment iC3b [94]. Therefore, despite the mentioned difficulties, the oral route is one of the most promising due to its good adhesion, broad spectrum of therapeutic regions, and easy operation [95].

Furthermore, oral administration of vaccines has immunological advantages because of the stimulation of mucosal and systemic responses [11]. Additionally, some studies have applied the use of YS as adjuvants to assist in the local response to vaccine application [96-98]. In the study by Bi *et al.* [97] the product AngelPW220 derived from *S. cerevisiae* containing β-glucan (≥30 %) and mannan oligosaccharides (MOS) (≥20 %) (Angel Yeast, Yichang, China) was tested. YS was added to the oral basal diet of Sanhuang chickens, which then received intraocular and intranasal immunization with a live NVD (Newcastle disease virus) vaccine [97]. In this specific study, it was observed that birds subjected to YS supplementation had significantly elevated serum levels of IL-4, IFN-γ, IL-6, and TNF-β compared to those that received the vaccine alone. These results indicated that YS can safely stimulate both Th1 and Th2 immune responses in animals [97,98]. Therefore, it is necessary to conduct further investigations into YS as a potential immunopotentiator and its combination with mucosal administration strategies.

## An innovative approach for biotechnological applications: yeast derivatives

Nanomedicine has emerged in recent decades as an auxiliary tool for improving the immune response against different antigens [99]. A study demonstrated that the use of nanoparticles in conjunction with β1,3-glucan decreased the levels of pro-inflammatory cytokines in supernatants of bone marrow dendritic cells cultured *in vitro*, and the immunomodulatory effect of this system was dependent on the size of the nanoparticles [100]. When comparing different diameters of NPY, it was observed that 355 nm glucan particles induced the secretion of IL-6 and TNF-α. This effect was not observed in smaller particles, suggesting that the immunomodulatory effect of NPY is associated with particle size, configuring an important parameter to ensure therapeutic efficacy in nanomedicine [101].

NPY has been studied for the treatment of chronic inflammatory diseases such as rheumatoid arthritis. Its use for carrying methotrexate (MTX) showed good biocompatibility and targeting macrophages, leading to the polarization of macrophages from the M1 to M2 type, in addition to reducing pro-inflammatory factors. Thus, the use of NPY may be a safe alternative strategy for clinical treatment to reduce inflammation and protect joints, enhancing the therapeutic effect [102].

One of the factors that impairs the induction of anti-cancer immune responses is the tumor microenvironment (TME) composition. However, the TME immunological profile can be manipulated by β-glucans particles that can modulate the TME by altering the phenotype of immunosuppressive cells [76,103]. In this sense, He *et al.* [16] observed that this system promotes the activation of bone marrow-derived macrophages, significantly contributing to the delay in tumor progression [16].

Silica associated with GP structure blocks protein charges and their thermal denaturation through “imprisonment in silica”. Thus, an ensilicated GP vaccine delivery system (GP + a layer of silica inside the hollow GP cavity) showed high immunogenicity, protecting vaccinated mice from lethal pulmonary infection by *Cryptococcus neoformans* [80]. These biomimetic and magnetic nanoparticles did not cause the production of pro-inflammatory mediators (IL-6 and TNF-α), formation of reactive oxygen species (ROS), or alteration in monocytes and dendritic cell viability [104].

The immunotherapeutic effect of a delivery system for carboxymethylated β- d -glucan (CMPTR) and iron oxide nanoparticles (IONPs) (CMPTR/IONPs) was evaluated in cultured bone marrow-derived macrophage cells and B16F10 melanoma from skin cancer patients, and led to reduced cancer cell migration [105]. The encapsulation of nanoparticles complexed with humic acid and iron (HA-Fe NPs) within glucomannan lipid particles (GMLPs) extracted from the yeast cell wall was efficient in tests carried out in vivo against the toxicity of aflatoxin B (AFB 1), presenting protective effect also against oxidative stress and histological changes in the liver and kidneys [106]. The use of gold nanoparticles (AuNPs) and platinum (PtNPs) complexed in yeast microcapsules seems to be efficient in activating immune responses and remodeling the tumor microenvironment, acting in synergy with chemodynamic therapy and photothermal therapy [107]. Attachment of β-glucan particles loaded with inflammasome inhibitor protein 3 (NLRP3) onto nanoparticle surface enabled precise and efficient targeting of cardiac ischemic/reperfusion injury and showed higher efficacy when compared to nanomicelle formulation, being approved by the Food Drugs Association (FDA) [108].

## Conclusions

Using yeast shells as carriers for therapeutic agents, proteins, and biomolecules, including nucleic acids, offers a unique combination of safety, low toxicity upon administration, and intrinsic potential as an adjuvant and modulator of the immune system. The exploration of various yeast-derived particles, such as GPs, WGPs, YCWPs, and others as YSs, reflects a versatility of use that can be tailored to optimize absorption and immunological response in target organisms.

In particular, applying YSs in oral formulations may allow for the induction of a systemic response through mucosal surfaces, which, for example, can be an effective strategy in the treatment and prevention of inflammatory diseases affecting the respiratory tract. As discussed in this article, there is a variety of methods available for the formulation of YSs alongside the elements of interest to be transported. The relative simplicity of these procedures may make them accessible and cost-effective alternatives in various applications.

However, it is important to emphasize that continuous advancements in research are necessary for a deeper understanding of the mechanisms of interaction and processing of YSs in target organisms, as well as the development of stabilisation techniques that ensure proper delivery and absorption of the transported compounds. Nevertheless, yeast shells present remarkable potential and versatility that may prove useful in optimizing biotechnological processes in search of solutions for medical conditions.
